# Phenotypic Effects of Wild-Type and Mutant SOD1 Expression in N9 Murine Microglia at Steady State, Inflammatory and Immunomodulatory Conditions

**DOI:** 10.3389/fncel.2019.00109

**Published:** 2019-04-09

**Authors:** Ana Rita Vaz, Sara Pinto, Catarina Ezequiel, Carolina Cunha, Luís A. Carvalho, Rui Moreira, Dora Brites

**Affiliations:** ^1^Research Institute for Medicines (iMed.ULisboa), Faculty of Pharmacy, University of Lisbon, Lisbon, Portugal; ^2^Department of Biochemistry and Human Biology, Faculty of Pharmacy, University of Lisbon, Lisbon, Portugal; ^3^Department of Pharmaceutical Chemistry and Therapeutics, Faculty of Pharmacy, University of Lisbon, Lisbon, Portugal

**Keywords:** amyotrophic lateral sclerosis, mutant SOD1^G93A^, microglia reactivity, inflammatory-associated microRNAs, glycoursodeoxycholic acid, vinyl sulfone

## Abstract

Accumulation of mutated superoxide dismutase 1 (mSOD1) in amyotrophic lateral sclerosis (ALS) involves injury to motor neurons (MNs), activation of glial cells and immune unbalance. However, neuroinflammation, besides its detrimental effects, also plays beneficial roles in ALS pathophysiology. Therefore, the targeting of microglia to modulate the release of inflammatory neurotoxic mediators and their exosomal dissemination, while strengthening cell neuroprotective properties, has gained growing interest. We used the N9 microglia cell line to identify phenotype diversity upon the overexpression of wild-type (WT; hSOD1^WT^) and mutated G93A (hSOD1^G93A^) protein. To investigate how each transduced cell respond to an inflammatory stimulus, N9 microglia were treated with lipopolysaccharide (LPS). Glycoursodeoxycholic acid (GUDCA) and dipeptidyl vinyl sulfone (VS), known to exert neuroprotective properties, were tested for their immunoregulatory properties. Reduced Fizz1, IL-10 and TLR4 mRNAs were observed in both transduced cells. However, in contrast with hSOD1^WT^-induced decreased of inflammatory markers, microglia transduced with hSOD1^G93A^ showed upregulation of pro-inflammatory (TNF-α/IL-1β/HMGB1/S100B/iNOS) and membrane receptors (MFG-E8/RAGE). Importantly, their derived exosomes were enriched in HMGB1 and SOD1. When inflammatory-associated miRNAs were evaluated, increased miR-146a in cells with overexpressed hSOD1^WT^ was not recapitulated in their exosomes, whereas hSOD1^G93A^ triggered elevated exosomal miR-155/miR-146a, but no changes in cells. LPS stimulus increased M1/M2 associated markers in the naïve microglia, including MFG-E8, miR-155 and miR-146a, whose expression was decreased in both hSOD1^WT^ and hSOD1^G93A^ cells treated with LPS. Treatment with GUDCA or VS led to a decrease of TNF-α, IL-1β, HMGB1, S100B and miR-155 in hSOD1^G93A^ microglia. Only GUDCA was able to increase cellular IL-10, RAGE and TLR4, together with miR-21, while decreased exosomal miR-155 cargo. Conversely, VS reduced MMP-2/MMP-9 activation, as well as upregulated MFG-E8 and miR-146a, while producing miR-21 shuttling into exosomes. The current study supports the powerful role of overexpressed hSOD1^WT^ in attenuating M1/M2 activation, and that of hSOD1^G93A^ in switching microglia from the steady state into a reactive phenotype with low responsiveness to stimuli. This work further reveals GUDCA and VS as promising modulators of microglia immune response by eliciting common and compound-specific molecular mechanisms that may promote neuroregeneration.

## Introduction

Amyotrophic lateral sclerosis (ALS) is a fatal motor neuron (MN) disease that affects both upper MNs, in the motor cortex, and lower MNs, in the brainstem and spinal cord. The major hallmark of this disease is the accumulation of intracellular protein inclusions in MNs, thought to be caused by mutations, protein damage such as oxidation, or protein seeding (Chiò et al., 2013; Robberecht and Philips, 2013). Among the several genes linked to ALS, there is evidence supporting a pathogenic role for Cu/Zn superoxide dismutase 1 (SOD1) in 20% of familial ALS cases and 3% of sporadic cases (Krüger et al., 2016). In fact, mutations in SOD1 are associated with a toxic gain of function that leads to protein misfolding and aggregation of the protein intracellularly (Rotunno and Bosco, 2013). More importantly, the uptake of misfolded and aggregated mutant SOD1 by other cells induces aggregation of endogenous mutant SOD1 and wild-type (WT) SOD1 protein (Münch et al., 2011; Sundaramoorthy et al., 2013). Small extracellular vesicles, here also designated as exosomes, have particular relevance as part of the cell-to-cell signaling mechanisms. Actually, once released, these vesicles can be taken up by nearby cells or travel long distances, affecting cellular function in either physiological or pathological ways (Sarko and McKinney, 2017). Misfolded and mutant SOD1 can be released *via* exocytosis, upon apoptosis or, as recently reported, inside exosomes (Gomes et al., 2007; Basso et al., 2013; Silverman et al., 2016). Once released into the extracellular space, mutant SOD1 activates microglia (Zhao et al., 2010) and we recently showed that the engulfment of exosomes released from mutant SOD1 MNs by microglia leads to the activation of inflammatory signaling pathways and loss of their phagocytic ability (Pinto et al., 2017). Despite decades of research and several studies pointing to SOD1 toxic function as the main player in ALS pathogenesis, the exact role of SOD1^WT^ and the impact of the mutated form in microglia function remains unclear. The relevance of microglia in the onset and progression of ALS is increasingly recognized and different polarized activated phenotypes were found in several models of ALS. In the majority of the studies using mutated SOD1 models, microglia overactivation was shown to contribute for ALS progression (Beers et al., 2006; Boillée et al., 2006). Two types of microglial activation have been considered, the classical M1 phenotype associated with the release of pro-inflammatory molecules and activation of receptors, and the M2 phenotype related with the secretion of anti-inflammatory mediators and growth factors, contributing to the repair and neuroprotection (Brites and Fernandes, 2015; Komine and Yamanaka, 2015). However, the latest knowledge points to the coexistence of different heterogeneous states and mixed phenotypes (Tang and Le, 2016; Pinto et al., 2017), and anti-inflammatory strategies have been replaced by the concept of active immunomodulation (Pena-Altamira et al., 2016). Actually, microglia activation was described as having both beneficial and injurious effects in ALS, depending on the relative prevalence of harmful and protective genes, on the ALS disease model and on the state of disease progression (Liao et al., 2012; Brites and Vaz, 2014; Gravel et al., 2016). In this sense, while the reduction of microgliosis was shown to slow ALS progression in the mutated SOD1 mice (Martínez-Muriana et al., 2016), reactive microglia was protective to MN degeneration in a mouse model of TDP-43 proteinopathy (Spiller et al., 2018), reinforcing the relevance of microglia reactivity and function in the ALS context.

Inflammatory-associated microRNAs (inflamma-miRNAs) are without doubt a new paradigm for understanding immunoregulation and inflammation. They showed to be important mediators of macrophages/microglia polarization and were found as part of microglia exosomal cargo, thus being able to modulate other cells (Alexander et al., 2015; Cardoso et al., 2016; Cunha et al., 2016; Fernandes et al., 2018). One of the miRNAs that gained particular attention in ALS is miRNA(miR)-155, already described in fALS and sALS patients (Koval et al., 2013), and also in the pre-symptomatic mutated superoxide dismutase 1 (mSOD1) mice, even before MN loss (Cunha et al., 2018), pointing this miRNA as a promising biomarker in ALS. Interestingly, targeting of miR-155 restored microglial proper functions in mSOD1 mice and prolonged mice survival (Butovsky et al., 2015), suggesting the benefits of molecules targeting miR-155 levels to be used as therapeutic strategies.

Currently, there are still no specific targets and effective therapies for ALS, due to the involvement of several multifactorial pathophysiological mechanisms. The only available treatments licensed by the Food and Drug Administration (FDA) are riluzole, since 1996, and the new compound, Edaravone (Abe et al., 2017; Cruz, 2018). Our previous studies have demonstrated that glycoursodeoxycholic acid (GUDCA), a conjugated bile acid, has antioxidant, anti-inflammatory and neuroprotective effects in a cellular model of mutant SOD1 neurodegeneration (Vaz et al., 2015). Lately, we also showed that dipeptidyl vinyl sulfone (VS), a small molecule with inhibitory cysteine protease activity, was able to prevent amyloid-β(Aβ)-induced microglia-inflammatory signaling pathways in the N9 microglia cell line, by inhibiting high mobility group box protein 1 (HMGB1) and interleukin(IL)-1β production (Falcão et al., 2017). Surprisingly, VS was also able to rescue miR-155 overexpression, thus indicating its potential in miR-155 immunoregulation.

Mice overexpressing human SOD1^WT^ have been used as controls, but some studies revealed the existence of abnormalities in the cerebellum (Afshar et al., 2017) and others that hastened the disease in G85R transgenic mice (Wang et al., 2009), highlighting that exaggerated expression of SOD1 is clearly not physiological. In fact, overexpression of both *SOD1* and *SOD1G93A* genes account for an elevation of SOD1 protein levels and changes in iron metabolism genes expression (Gajowiak et al., 2015). However, in the N9 murine microglia, SOD1 overexpression decreased the release of tumor necrosis factor α (TNF-α) and IL-6 upon stimulation with lipopolysaccharide (LPS; Dimayuga et al., 2007), and that of mutant SOD1 in BV-2 microglial cell lines increased TNF-α secretion and their neurotoxic potential (Liu et al., 2009). It is thus essential to significantly expand our knowledge on the molecular mechanisms involved in ALS microglia dysfunctional properties to target key steps with novel drugs. In the present study, we assessed the inflammatory phenotype diversity of N9 murine microglia upon transduction to promote the overexpression of human SOD1, WT (hSOD1^WT^) or mutated G93A (hSOD1^G93A^) protein, relatively to naïve cells. To have a better insight on how such transduction may differently compromise the microglia response to an inflammatory stimulus, we treated these cells with LPS and evaluated a set of inflammatory and anti-inflammatory mediators, as well as inflamma-miRNAs. Finally, we assessed the potential immunoregulatory properties of GUDCA and VS.

Data revealed that both hSOD1^WT^ and hSOD1^G93A^ overexpression in N9-microglia induced morphological shape alterations and reduction of M2 markers, compatible with an activated cell. Major inflammatory genes were only increased in the hSOD1^G93A^ cells, which released exosomes enriched in miR-155 and miR-146a, as well as in HMGB1 and SOD1 mRNAs. Interestingly, transduced cells showed a defective stimulus-response to LPS, in contrast with the naïve cells that presented exacerbated inflammatory markers. We also provide evidence that GUDCA and VS exert immunomodulatory properties over the hSOD1^G93A^ microglia, but that their benefits are mediated by molecular pathways that are specific of each compound. Thus, we may conclude that depending on the key molecular cascades to be modulated towards neuroprotection, GUDCA and VS can be effective therapeutic strategies to restore microglial function in ALS.

## Materials and Methods

### N9 Cell Line Transduction: Lentiviral Production and Generation of N9 Cells Expressing hSOD1^WT^-GFP and hSOD1^G93A^-GFP Protein

**Figure 1:**
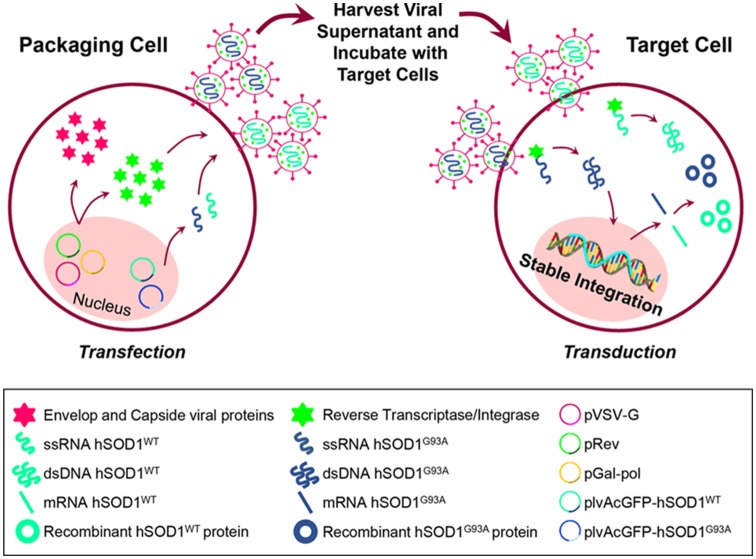
Schematic representation of cell transfection for lentiviral production and further generation of N9 cells expressing hSOD1^WT^-green fluorescent protein (GFP) and hSOD1^G93A^-GFP protein. HEK293T cells were incubated with the packaging plasmids pGal-pol and pRev, the envelop plasmid pVSV-G and the lentiviral expression vectors plvAcGFP-hSOD1^WT^ and plvAcGFP-hSOD1^G93A^, in order to produce lentivirus carrying single-strand RNAs (ssRNA) containing the sequence for human superoxide dismutase 1 wild-type (WT; hSOD1^WT^), and carrying the G93A mutation (hSOD1^G93A^). Cell supernatants were collected and incubated with N9 naïve cells, then reverse transcriptase converted the ssRNA into double-strand RNA (dsRNA) that entered the cell nucleus and stably integrated the genome.

We used the N9 microglial cell line (Cunha et al., 2016; Pinto et al., 2017). These cells are reported to present features similar to microglia in primary cultures, such as phagocytosis and inflammation-related features (Righi et al., 1989; Bruce-Keller et al., 2000; Fleisher-Berkovich et al., 2010). Lentiviral particles were produced by co-transfections of HEK293T cells with the packaging plasmids pGal-pol and pRev, the envelope plasmid pVSV-G and the lentiviral expression vectors plvAcGFP-hSOD1wt/plvAcGFP-hSOD1G93A (plasmids #27138 and #27142, respectively, Addgene, Cambridge, USA; Stevens et al., 2010), at a ratio of 3:2:1:4, using X-tremeGene HP, according to the manufacturer’s instructions (Roche, Mannhein, Germany; Simões et al., 2015; Pereira et al., 2016). Twenty-four hours after transfection, cell media was changed. Supernatants containing lentiviral particles were collected after 48 and 72 h, filtered using a 0.22 μm sterile filter and stored at −80°C. To stably overexpress hSOD1^WT^ and hSOD1^G93A^ in N9 microglia cell line, cells were seeded in 6-well plates at a density of 3 × 10^5^ cells/well and, 24 h after plating, cells were transduced by adding the supernatants containing lentiviral particles. Media was changed 4–5 times to eliminate all the lentiviral particles (Simões et al., 2015; Pereira et al., 2016). Stable cell lines were purified by cell sorting of green fluorescent protein (GFP) expression (BDFACSAria, BD Biosciences) and the percentage of GFP-positive cells was monitored in the GUAVA flow cytometer. The percentage of GFP-expressing cells was >80% in all experiments (Figure 1). Cells were maintained in Roswell Park Memorial Institute (RPMI) medium supplemented with 10% fetal bovine serum (FBS), 1% L-glutamine and 1% Penicillin/Streptomycin. For each experiment, cells were plated at a concentration of 1 × 10^5^ cells/ml (Cunha et al., 2016). RPMI was purchased from Sigma-Aldrich (St. Louis, MO, USA). FBS, L-glutamine and Penicillin/Streptomycin were purchased from Biochrom AG (Berlin, Germany).

### N9 Cells Incubation

After 24 h of plating, cellular media was changed and either hSOD1^WT^ and hSOD1^G93A^ cells were maintained for 48 h, treated or not with LPS (300 ng/ml). Non-transduced N9 cells (naïve) were used as controls, and also treated or not with LPS. In another set of experiments, hSOD1^G93A^ cells were incubated with GUDCA (50 μM) or with VS (10 μM), also during 48 h.

### Exosome Isolation

Exosomes were obtained from the extracellular media of N9 naïve, hSOD1^WT^ and N9 hSOD1^G93A^ cells, according to our previous publication (Pinto et al., 2017). Briefly, after the time of incubation, extracellular media was centrifuged at 1,000 *g* for 10 min, to remove cell debris. Then, the supernatant was transferred to a different tube and centrifuged at 16,000 *g* for 1 h to pellet microvesicles. The recovered supernatant was filtered in a 0.22 μm pore filter, transferred to an ultracentrifuge tube and centrifuged at 100,000 *g* for 2 h, to pellet exosomes. Afterward, the pellet of exosomes was resuspended in phosphate-buffered saline (PBS) and centrifuged one last time at 100,000 *g* for 2 h, in order to wash the pellet. All the procedure was performed at 4°C. Exosome pellet was resuspended in lysis buffer for further RNA isolation.

### Negative-Staining Transmission Electron Microscopy

Exosomes were visualized by transmission electron microscopy (TEM); for negative staining TEM, 10 μL of samples were mounted on Formvar/carbon film-coated mesh nickel grids (Electron Microscopy Sciences, Hatfield, PA, USA) and left standing for 2 min. The liquid in excess was removed with filter paper, and 10 μL of 1% uranyl acetate was added on to the grids and left standing for 10 s, after which, the liquid in excess was removed with filter paper. Visualization was carried out on a JEOL JEM 1400 TEM at 120 kV (Tokyo, Japan). Images were digitally recorded using a CCD digital camera Orious 1100W Tokyo, Japan at the HEMS/i3S of the University of Porto.

### Western Blot

Cells were collected in Cell Lysis Buffer and protein concentration was determined using a protein assay kit (Bio-Rad, Hercules, CA, USA) according to manufacturer’s specifications. Then, 50 μg of protein were separated in a 12% polyacrylamide electrophoresis gel (SDS-PAGE) and transferred to a nitrocellulose membrane. After blocking with 5% (w/v) nonfat milk solution, membranes were incubated with the following primary antibodies: rabbit anti-SOD1 (1:500), mouse anti-GFP (1:1,000 both from Santa Cruz Biotechnology^®^) or anti-β-actin (1:5,000), from Sigma, all diluted in 5% (w/v) BSA overnight at 4°C, followed by the secondary antibodies goat anti-rabbit or goat anti-mouse, respectively, both HRP-linked (1:5,000 Santa Cruz Biotechnology^®^) diluted in blocking solution. Chemiluminescence detection was performed by using Western Bright^TM^ Sirius (K- 12043-D10, Advansta, Menlo Park, CA, USA) and bands were visualized in the ChemiDoc^TM^ XRS System (Bio-Rad; Vaz et al., 2015). In parallel, we performed western blot analysis in isolated exosomes to evaluate the expression of Alix, Flotillin-1 and CD63 by using the same procedure, 20 μg of total protein and specific antibodies (mouse anti-Alix, Cell Signaling, mouse anti-flotillin-1, BD Biosciences, goat anti-CD63, Santa Cruz Biotechnology). In this case, we used Amido Black staining as the loading control.

### Quantitative Real Time-PCR

Total RNA was extracted from N9 microglia using TRIzol^®^ (LifeTechnologies, Carlsbad, CA, USA), according to manufacturer’s instructions. RNA inside exosomes was extracted using miRCURY Isolation Kit—Cell (#300110, Exiqon, Vedbaek, Denmark). Total RNA was quantified using Nanodrop ND-100 Spectrophotometer (NanoDrop Technologies, Wilmington, DE, USA) and conversion to cDNA was performed with GRS cDNA Synthesis Master Mix (GRiSP, Porto, Portugal). Quantitative Real Time-PCR (qRT-PCR) was performed on a QuantStudio 7 Flex Real-Time PCR System (Applied Biosystems) using an Xpert Fast Sybr Blue (GRiSP). qRT-PCR was accomplished under optimized conditions: 50°C for 2 min followed by 95°C for 2 min and finally 50 cycles at 95°C for 5 s and 62°C for 30 s. In order to verify the specificity of the amplification, a melt-curve analysis was performed, immediately after the amplification protocol. Non-specific products of PCR were not found in any case. Results were normalized to β-actin and expressed as fold change. The sequences used for primers are represented in Supplementary Table S1. For miRNA analysis, conversion of cDNA was achieved with the universal cDNA Synthesis Kit (#203301, Exiqon; Cunha et al., 2018). The Power SYBR^®^ Green PCR Master Mix (Applied Biosystems) was used in combination with predesigned primers (Exiqon), represented in Supplementary Table S1, using SNORD110, U6 and RNU1A1 as reference genes. The reaction conditions consisted of polymerase activation/denaturation and well-factor determination at 95°C for 10 min, followed by 50 amplification cycles at 95°C for 10 s and 60°C for 1 min (ramp-rate 1.6°/s). Relative mRNA and miRNA concentrations were calculated using the ΔΔCT equation and quantification of target miRNAs was made in comparison to the geometric average of the three reference genes. In addition, we used the synthetic RNA template spike-in (UniSp6) as a positive control to ensure the quality of the reaction and subsequent evaluations. All samples were measured in duplicate.

### Assessment of Gelatinases (MMP-2 and MMP-9) by Gelatin Zymography

Activities of MMP-2 and MMP-9 were determined in the N9 extracellular media, either alone or after incubation with LPS, GUDCA or VS, by performing a SDS-PAGE zymography in 0.1% gelatin-10% acrylamide gels, under non-reducing conditions (Silva et al., 2010). Briefly, after electrophoresis, the gels were washed for 1 h in a solution containing 2.5% Triton-X-100 to remove SDS and to renature the MMP species in the gel and then incubated at 37°C to induce gelatin lysis (buffer: 50 mM Tris pH 7.4, 5 mM CaCl_2_, 1 μM ZnCl_2_) overnight. Gels were then stained with 0.5% Coomassie Brilliant Blue R-250 (Sigma-Aldrich) and destained in 30% ethanol/10% acetic acid/H_2_O (v/v). Gelatinase activity, detected as a white band on a blue background, was measured using the Image Lab^TM^ analysis software (Bio-Rad).

### Quantification of Nitrite Levels

Levels of nitric oxide (NO) were estimated by measuring the concentration of nitrites (${\text{NO}}_{2}^{-}$), a product of NO metabolism, in the extracellular media of N9 cells. Extracellular medium, free from cellular debris, was mixed with Griess reagent [1% (w/v) sulfanilamide in 5% H_3_PO_4_ and 0.1% (w/v) N-1 naphtylethylenediamine, all from Sigma-Aldrich, in a proportion of 1:1 (v/v)] in 96-well tissue culture plates for 10 min in the dark, at room temperature (Vaz et al., 2015). The absorbance at 540 nm was determined using a microplate reader (Bio-Rad Laboratories; Hercules, CA, USA). A calibration curve was used for each assay. All samples were measured in duplicate.

### Statistical Analysis

Results of at least three independent experiments were expressed as mean ± SEM. Comparisons between N9 naïve and N9 naïve incubated with LPS were made using unpaired two-tailed Student’s *t*-test. Comparisons between the different groups were made by one-way ANOVA followed by multiple comparisons Bonferroni *post hoc* correction using GraphPad Prism 7 (GraphPad Software, San Diego, CA, USA). *P*-values of 0.05 were considered statistically significant.

## Results

### Overexpression of Human WT and Mutant SOD1 in N9 Microglia Does Not Produce Significant Alterations in Cellular Viability or Phagocytic Ability and Neither in the Number of Cell-Derived Exosomes

**Figure 2:**
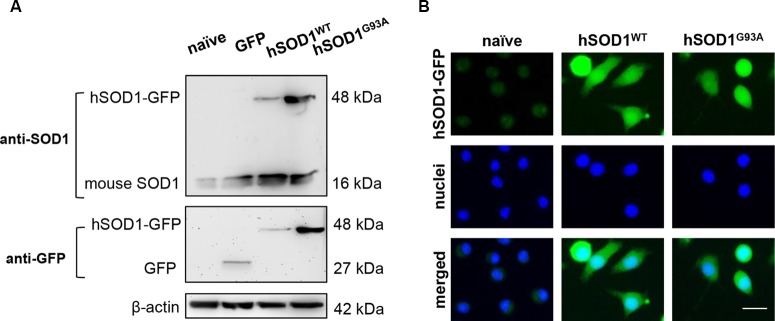
Transduced N9 microglia efficiently express the human SOD1 protein. The presence of hSOD1 WT and carrying the G93A mutation (hSOD1^WT^ and hSOD1^G93A^, respectively), coupled with the green fluorescent protein tail (hSOD1-GFP), as well as the expression of GFP was confirmed by Western blot and immunocytochemistry. **(A)** Western blot analysis indicates the presence of mouse SOD1, as well as hSOD1-GFP at a higher weight, while GFP expression in only observed for cells transduced with GFP not fused with hSOD1. Representative results from one blot are shown and β-actin was used as a loading control. Samples ran in the same gel at the same conditions. **(B)** Expression of hSOD1-GFP was confirmed by immunocytochemistry (green fluorescence). Nuclear staining was achieved with Hoechst dye (in blue). Representative results of one experiment are shown. Scale bar represents 20 μm.

To achieve SOD1 overexpression in microglia, N9 naïve cells were transduced with hSOD1^WT^ and hSOD1^G93A^ coupled with a GFP tail, as detailed in the methods section, which allowed us to monitor protein expression over time. As previously observed in our group, Western blot analysis showed the presence of mouse SOD1 protein expression at 16 kDa in all samples (Vaz et al., 2015). In N9 hSOD1^WT^ and hSOD1^G93A^ microglia, hSOD1 expression was verified at ~48 kDa, consistent with the weight of hSOD1 coupled with the GFP tail (Figure 2A). In addition, the 27 kDa band corresponding to GFP alone (indicated in additional supporting information) was only detected in cells transduced with GFP alone upon incubation with anti-GFP antibody. To note that, although appearing slightly higher in N9 hSOD1^WT^ microglia, we did not find significant differences between hSOD1^WT^ and hSOD1^G93A^ expression, also confirmed by the percentage of GFP-positive cells checked routinely in the GUAVA flow cytometer, as well as by immunocytochemistry (Figure 2B). We additionally evaluated cellular morphology, phagocytic ability and cell viability to assess the impact of hSOD1 transduction in these cells. However, no differences were found between hSOD1^WT^ and hSOD1^G93A^ cells (Supplementary Figure S1).

**Figure 3:**
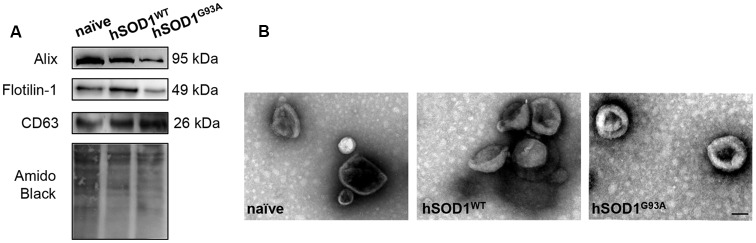
Exosomes released by N9 cells, either naïve or expressing hSOD1^WT^ or hSOD1^G93A^ show similar markers, size and shape. Exosomes were isolated from the extracellular media of N9 cells, either naïve or overexpressing human hSOD1^WT^ and hSOD1^G93A^, as described in methods. **(A)** Western blot analysis indicates the presence of common exosome markers Alix, Flotillin-1, and CD63. **(B)** Representative images obtained by negative-staining transmission electron microscopy (TEM) of exosomes are depicted evidencing cup shape morphology and that the majority of vesicles have a diameter of close to 100 nm. Scale bar represents 100 nm.

In our previous work, we demonstrated that neuronal exosomes from hSOD1^G93A^ cells carry a content of inflamma-miRNAs similar to the cells of origin, facilitating the shuttle of miR-124 from neuron to microglia, while induce phenotypic alterations and cellular reactivity in recipient microglia cells (Pinto et al., 2017). Therefore, in the current study, we isolated exosomes from N9, either naïve or overexpressing hSOD1^WT^ or hSOD1^G93A^. We have confirmed the presence of Alix, Flotillin-1 and the tetraspanin CD63 in exosomal lysates (Figure 3A). Diameter exosome size was ~100 nm and cup-shape morphology was obtained by TEM (Figure 3B).

### N9 hSOD1^WT^ Microglia Show Downregulated Anti-/Pro-inflammatory Genes, While N9 hSOD1^G93A^ Cells Express Upregulated MFG-E8, RAGE and Pro-inflammatory Genes, and Release Exosomes Enriched in HMGB1 and SOD1

**Figure 4:**
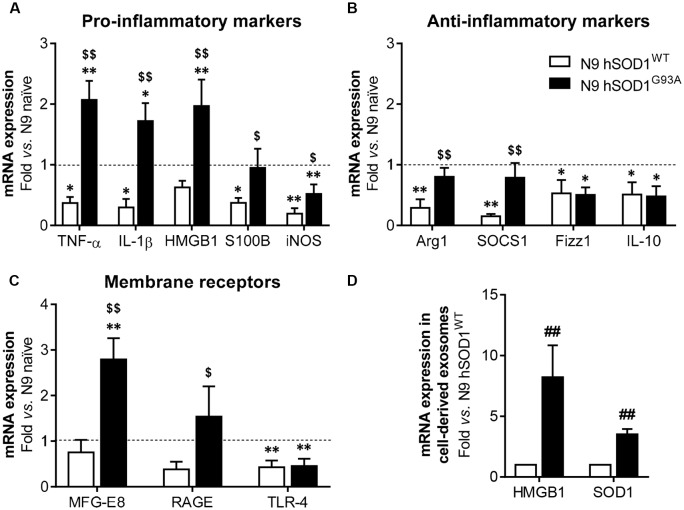
N9 hSOD1^G93A^ microglia have a pro-inflammatory phenotype with an exacerbated MFG-E8 and RAGE gene expression, and originate exosomes enriched in HMGB1 and SOD1. Cellular mRNA expression of pro-inflammatory markers **(A)**, anti-inflammatory markers **(B)** and membrane surface receptors **(C)** was assessed by quantitative Real-Time PCR (qRT-PCR). The dashed line represents the average value (N9 naïve cells). **(D)** mRNA expression was also evaluated in exosomes by qRT-PCR and N9 hSOD1^WT^ cells were used as a control. Results are mean (± SEM) from at least three independent experiments. *p < 0.05 and **p < 0.01 vs. N9 naïve microglia; ^$^*p* < 0.05 and ^$$^*p* < 0.01 vs. N9 hSOD1^WT^ microglia; one-way ANOVA (Bonferroni *post hoc* correction). ^##^*p* < 0.01 vs. N9 hSOD1^WT^ cells; unpaired Student’s *t*-test (two-tailed).

Although SOD1 mutations are reported in fALS cases (Barber and Shaw, 2010), the effect of mutant SOD1^G93A^ specifically in microglia is not completely clarified. Recently, reactive microglia evidenced to exert neuroprotective functions in a mouse model of TDP-43 proteinopathy (Spiller et al., 2018), highlighting the beneficial role that microglia activation may also have in neurodegenerative diseases. To explore the reactive signature of WT and mutant SOD1 in N9 microglia, we evaluated the gene expression of specific inflammatory markers in naïve and in transduced cells overexpressing hSOD1. When compared to naïve cells, N9 hSOD1^WT^ microglia showed decreased levels of pro-inflammatory markers (usually associated to the M1 phenotype, TNF-α/IL-1β/S100B/iNOS), as well as anti-inflammatory markers (related to the M2 phenotype, Arg1/SOCS1/Fizz1/IL-10; Figures 4A,B). Although some anti-inflammatory markers were similar to naïve cells (Arg1 and SOCS1) or reduced in a similar way to those observed in N9 hSOD1^WT^ microglia (Fizz1 and IL-10), N9 hSOD1^G93A^ cells showed an increase in the pro-inflammatory markers that were studied. More importantly, some of these markers (TNF-α, IL-1β and HMGB1) were not only above the levels obtained in N9 hSOD1^WT^ cells, but also above those of naïve cells, which indicate a stressed microglia that may contribute to either a depressed or exacerbated response, depending on the inflammatory stimulus (Figures 4A,B). Since we did not find significant differences between the levels of such markers in N9 transduced with GFP alone (Supplementary Figure S2), we may assume that the changes observed are due to the presence of hSOD1^WT^ or hSOD1^G93A^ and not because of the lentiviral infection. Moreover, increased expression of membrane surface receptors, like MFG-E8 and RAGE, was only evident in N9 hSOD1^G93A^ microglia, when compared to N9 hSOD1^WT^ microglia, while TLR-4 expression was reduced in both cells (Figure 4C). Interestingly, we observed that exosomes may also be involved in the propagation of the alarmin HMGB1 and the SOD1 protein, whose gene levels were found elevated in N9 hSOD1^G93A^ microglia, thus contrasting with N9 hSOD1^WT^ cells (Figure 4D). This result is consistent with previous findings where SOD1 was described to be transported into the extracellular media in exosomes and further collected by other cells (Silverman et al., 2016). This finding suggests that mutated microglia also contribute to chronic inflammatory status through the transport of HMGB1 in their released exosomes, never described to be carried by microglial-derived exosomes.

### N9 hSOD1^WT^ Microglia Show a Specific miR-146a Upregulation Not Recapitulated in Their Exosomes, While Those From hSOD1^G93A^ Cells Are Unique as Mediators of Neuroinflammation by Their Elevated miR-155 and miR-146a Cargo

**Figure 5:**
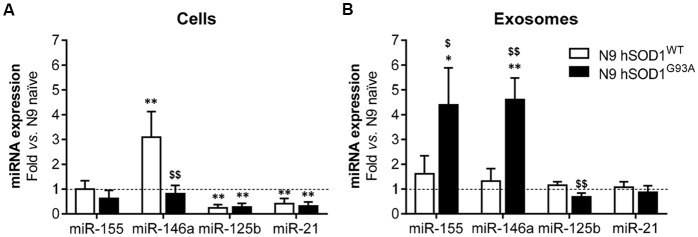
N9 hSOD1^WT^ microglia highly express miR-146a, while N9 hSOD1^G93A^ cells actively package miR-155 and miR-146a into their exosomes. Inflammatory microRNAs (miRNA, miR) expression in both cells **(A)** and exosomes **(B)** were analyzed by qRT-PCR. N9 naïve cells were used as a control and the dashed line represents the average value. Results are mean (± SEM) from at least three independent experiments. *p < 0.05 and **p < 0.01 vs. N9 naïve microglia; ^$^*p* < 0.05 and ^$$^*p* < 0.01 vs. N9 hSOD1^WT^ microglia; one-way ANOVA (Bonferroni *post hoc* correction).

Inflamma-miRNAs are reported to have a modulatory role in microglia activation (Cardoso et al., 2012; Saba et al., 2012) and to circulate as part of exosomal cargo (Alexander et al., 2015; Fernandes et al., 2018). In this study, we were mainly interested in assessing for the first time the differential expression of miR-155, miR-146a, miR-125b and miR-21 in N9 cells transduced with WT and mutant SOD1 relatively to the naïve ones, which are known to mediate macrophage polarization (Essandoh et al., 2016), CNS inflammation (Su et al., 2016) and to be expressed in the spinal cord of SOD1^G93A^ mice at the symptomatic stage (Cunha et al., 2018). Decreased levels of miR-125b and miR-21, when compared with the naïve cells (as well as cells transduced with GFP, Supplementary Figure S2), were found in WT and mutated microglia (Figure 5A), and may account to a compromised response to an inflammatory stimulus (Tili et al., 2007; Parisi et al., 2016), thus turning microglia more susceptible to activation (Barnett et al., 2016; Cardoso et al., 2016). Regarding the pro-inflammatory miR-155 and its negative regulator miR-146a, only the last one was increased in N9 hSOD1^WT^ microglia. While upregulated levels of miR-146a in hSOD1^WT^ cells may act as a negative regulator of miR-155 increase, thus accounting for their normal content in exosomes, selective release of miR-155 and miR-146a from hSOD1^G93A^ N9 cells into exosomes (Figure 5B) may drive disease progression in the mSOD1 ALS model. MiRNAs that are overrepresented in exosomes in comparison to levels in the cell indicate a selective exported mechanism (Bell and Taylor, 2017).

### Overexpression of Human WT and Mutant SOD1 in N9 Microglia Leads to a Defective Inflammatory Gene Response to LPS Stimulation, With an Increased miR-155 Sorting in Exosomes From WT Cells

It was demonstrated that SOD1^WT^ overexpression decreases ROS production and reduces neurotoxic inflammatory markers, even in the presence of LPS (Dimayuga et al., 2007). Therefore, we thought that would be interesting to further explore differences in hSOD1 microglia reactivity towards LPS treatment relatively to naïve cells, to better understand microglial behavior when facing an inflammatory environment in the context of ALS. As indicated in Table 1, incubation of naïve cells with LPS induced an activated phenotype with increased TNF-α, HMGB1 and S100B, but not of IL-1β or iNOS gene expression. Upregulation of Fizz1 and MFG-E8 was notorious, together with increased extracellular MMP-9 activation and NO production. Curiously, we found a decrease in the gene expression of S100B and RAGE. Stimulation with LPS also upregulated miR-155, miR-146a and miR-21, and downregulated miR-125b in cells. Interestingly, they were all found reduced in respective cell-derived exosomes, with the exception of miR-146a. However, either hSOD1^WT^ or hSOD1^G93A^ cells revealed lower expression of pro- and anti-inflammatory genes upon LPS challenge, than naïve N9 microglia.

**Table 1:** Differences in gene and miRNA expression, as well as in soluble factors, after exposure of naïve and transduced N9 microglia to lipopolysaccharide (LPS).

Markers	N9 naïve with LPS (fold change vs. N9 naïve)	N9 hSOD1^WT^ with LPS (fold change vs. N9 naïve with LPS)	N9 hSOD1^G93A^ with LPS (fold change vs. N9 naïve with LPS)
**Genes**			
**Pro-inflammatory markers**			
TNF-α	10.56 ± 3.87^++^	0.45 ± 0.10**	0.49 ± 0.21*
IL-1β	8.03 ± 5.05	0.74 ± 0.33	0.40 ± 0.12
HMGB1	1.69 ± 0.28^++^	0.56 ± 0.16**	0.38 ± 0.06**
S100B	0.40 ± 0.12^++^	1.14 ± 0.41	0.34 ± 0.12^$^
iNOS	1.56 ± 0.68	0.34 ± 0.15**	0.20 ± 0.04**
**Anti-inflammatory markers**			
Arg1	1.41 ± 0.45	0.33 ± 0.11**	0.23 ± 0.06**
SOCS1	0.31 ± 0.09^++^	0.46 ± 0.12**	0.51 ± 0.14**
Fizz1	7.40 ± 1.83^++^	0.33 ± 0.13**	0.26 ± 0.06**
IL-10	3.10 ± 1.75	0.32 ± 0.15**	0.29 ± 0.06**
**Receptors**			
MFG-E8	3.41 ± 0.99^+^	0.40 ± 0.17**	0.44 ± 0.12**
RAGE	0.46 ± 0.26^+^	0.46 ± 0.18**	0.31 ± 0.11**
TLR4	2.00 ± 0.65	2.97 ± 1.42	0.40 ± 0.10^$^
**Soluble factors**			
MMP-9	2.23 ± 0.34^++^	1.66 ± 0.42	1.16 ± 0.14
MMP-2	1.20 ± 0.15	0.97 ± 0.06	1.04 ± 0.14
NO	2.31 ± 0.23^+^	3.68 ± 1.25	2.96 ± 0.71
**MicroRNAs**			
miR-155	6.28 ± 1.95^++^	0.41 ± 0.15**	0.40 ± 0.19*
miR-146a	5.34 ± 1.10^++^	0.44 ± 0.17**	0.35 ± 0.15**
miR-125b	0.25 ± 0.12^++^	0.37 ± 0.11*	1.18 ± 0.38^$$^
miR-21	5.01 ± 1.66^+^	2.04 ± 0.44*	0.63 ± 0.24^$$^
**In exosomes**			
miR-155	0.34 ± 0.34^+^	7.88 ± 2.82*	0.30 ± 0.12^$^
miR-146a	1.25 ± 0.36	1.04 ± 0.30	1.02 ± 0.57
miR-125b	0.48 ± 0.07^++^	1.39 ± 0.52	0.49 ± 0.15
miR-21	0.36 ± 0.11^++^	2.53 ± 1.60	0.50 ± 0.20

Results are expressed as fold change (MEAN ± SEM) from at least three independent experiences for LPS-treated naïve cells vs. non-treated N9 naïve cells and for LPS-treated N9 hSOD1^WT^ and N9 hSOD1^G93A^ microglia vs. LPS-treated naïve cells. Comparisons between N9 naïve and N9 naïve with LPS were made using an unpaired two-tailed Student’s *t*-test. Comparisons between the three different groups (N9 naïve with LPS, N9 hSOD1^WT^ with LPS and N9 hSOD1^G93A^ with LPS) were made by One-way ANOVA followed by multiple comparisons Bonferroni *post hoc* correction. ^+^*p* < 0.05 and ^++^*p* < 0.01 vs. N9 naïve microglia; *p < 0.05 and **p < 0.01 vs. N9 naïve with LPS; ^$^*p* < 0.05 and ^$$^*p* < 0.01 vs. N9 hSOD1^WT^ with LPS.

It is important to note that GFP-transduced cells were able to respond to LPS in a similar manner to naïve N9 in terms of pro- or anti-inflammatory markers we used, as well as in miRNAs expression (Supplementary Figure S2), indicating that the differences in transduced cells do not derive from lentiviral transduction or GFP expression. In a similar way, MFG-E8 and RAGE mRNA levels were found downregulated in both transduced cells, in comparison with the LPS-treated naïve ones, while no significant differences were obtained for extracellular MMP-9, MMP-2 and NO levels. The same defective pattern was observed for inflamma-miRNAs, though an increase of miR-21 was observed in LPS-treated hSOD1^WT^ cells and of miR-125b in hSOD1^G93A^ transduced microglia. To highlight, however, that the selective exported mechanism of miR-155 into exosomes was notorious in the hSOD1^WT^ cells, contrasting with its low expression in mSOD1 cells and their exosomes after stimulation with LPS, further attesting their increased inability to develop an inflammatory response against damage.

### Mitigation of Pro-inflammatory Genes and Regulation of Inflamma-miRNAs in N9 hSOD1^G93A^ Microglia by GUDCA and VS Is Mediated by Common and Compound-Specific Molecular Mechanisms

**Figure 6:**
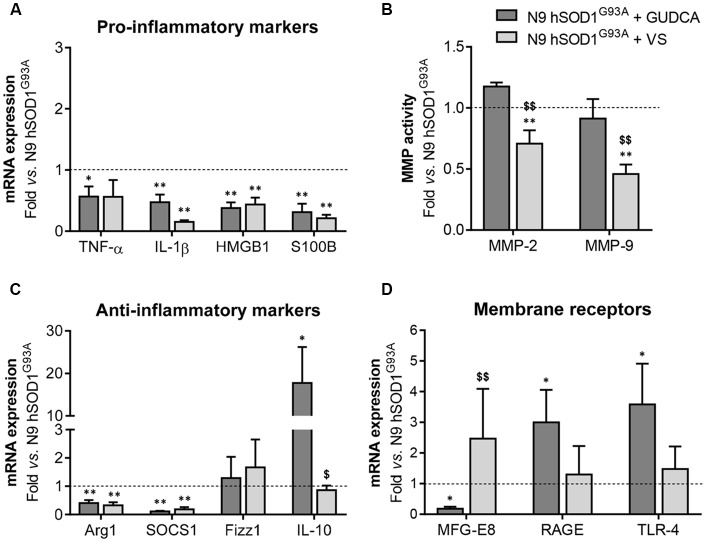
Pro-inflammatory markers in N9 hSOD1^G93A^ microglia are downregulated by glycoursodeoxycholic acid (GUDCA) and dipeptidyl vinyl sulfone (VS), with GUDCA specifically upregulating IL-10, RAGE and TLR-4 genes, and VS promoting the increase of MFG-E8 and the decrease of MMP-2 and MMP-9 activation. N9 hSOD1^G93A^ microglia were incubated with 50 μM of GUDCA or 10 μM of VS for 48 h. Cellular mRNA expression of pro-inflammatory markers **(A)**, anti-inflammatory markers **(C)** and membrane surface receptors **(D)** was evaluated by qRT-PCR. **(B)** Activation of MMP-2 and MMP-9 was assessed by gelatin zymography assay. The intensity of the bands was quantified using computerized image analysis (Image Lab^TM^ software). Non-treated mutant cells were used as control and the dashed line represents average values. Results are mean (± SEM) from at least three independent experiments. *p < 0.05 and **p < 0.01 vs. N9 hSOD1^G93A^ microglia; ^$^*p* < 0.05 and ^$$^*p* < 0.01 vs. N9 hSOD1^G93A^ + GUDCA; one-way ANOVA (Bonferroni *post hoc* correction).

**Figure 7:**
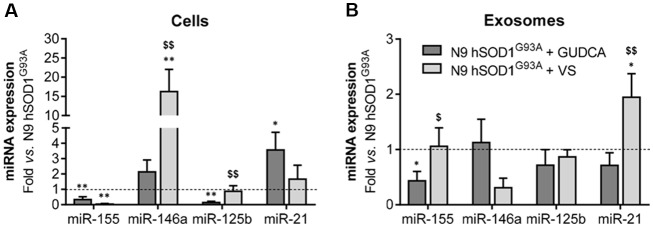
Treatment of N9 hSOD1^G93A^ microglia with glycoursodeoxycholic acid (GUDCA) and dipeptidil vinyl sulfone (VS) decreases miR-155, with GUDCA specifically upregulating miR-21 in cells, and VS promoting the increase of miR-146a in cells and of miR-21 in exosomes. After 48 h incubation of N9 hSOD1^G93A^ microglia with 50 μM of GUDCA or 10 μM of VS, inflammatory microRNAs (miRNA, miR) in both cells **(A)** and exosomes **(B)** were analyzed by qRT-PCR. Non-treated mutant cells were used as control and the dashed line represents the average values. Results are mean (± SEM) from at least three independent experiments. *p < 0.05 and **p < 0.01 vs. N9 hSOD1^G93A^ microglia; ^$^*p* < 0.05 and ^$$^*p* < 0.01 vs. N9 hSOD1^G93A^ + GUDCA; one-way ANOVA (Bonferroni *post hoc* correction).

The alterations observed in the expression of pro- and anti-inflammatory-associated genes and miRNAs, as well as the inability to mount an inflammatory response when facing an inflammatory stimulus in the microglia transduced with hSOD1^G93A^, led us to test two different compounds described as immunomodulators in glial cells (Fernandes et al., 2007; Falcão et al., 2017). Neither of them caused identifiable changes in cell viability (Supplementary Figure S3) and both were similarly efficient in decreasing the expression of genes associated to a pro-inflammatory response, such as TNF-α, IL-1β, HMGB1 and S100B (Figure 6A). Interestingly, we found that MMP-2 and MMP-9 activation was merely downregulated by VS in hSOD1^G93A^ cells (Figure 6B), supporting its specificity over that of GUDCA to inhibit these pro-inflammatory mediators. This may have therapeutic relevance since the elevation of MMP-9/-2 expression was observed in the spinal cord of SOD1^G93A^ mice (Fang et al., 2010), and that of MMP-9 in NSC-34 MNs overexpressing hSOD1^G93A^ (Vaz et al., 2015). Though that both VS and GUDCA showed to reduce the expression of the anti-inflammatory associated genes Arg1 and SOCS1, only GUDCA significantly enhanced the expression of IL-10 mRNA (Figure 6C), revealing its potential to counteract inflammation whenever it is exacerbated. Unique potential therapeutic benefits of VS were also found for its ability in increasing the gene expression of MFG-E8 (Figure 6D), a protein that has been described as promoting microglia phagocytosis (Lei et al., 2013). Further studies should elucidate whether activation of RAGE and TLR-4 by GUDCA, as we here observed, will have benefits in restoring the immune response of hSOD1^G93A^ cells. Additionally, both VS and GUDCA showed to be equally efficient in decreasing intracellular miR-155, while miR-21 was selectively enhanced by GUDCA and miR-146a by VS (Figure 7A), again reinforcing their action through distinct pathways. Sorting into exosomes was also different considering that GUDCA was again effective in sustaining dissemination of miR-155 and inflammation by exosomes released from hSOD1^G93A^ cells. In addition, exosome-shuttling of miR-21, indicated to have protective effects against oxidative stress (Shi et al., 2018), merely occurred by VS treatment (Figure 7B).

These novel data provide clear evidence of the beneficial contribution of VS and GUDCA as immunoregulatory compounds in the hSOD1^G93A^ N9 microglia, by sharing similar modulatory properties, but overall by additionally counteracting distinct inflammatory signaling pathways.

## Discussion

The role of glial cells in neurodegenerative diseases, particularly in ALS, has been thoroughly investigated and discussed, with evidences that these cells are key contributors to the neuroinflammation processes and disease progression (Lasiene and Yamanaka, 2011). In this study, we focused on microglia since they have a deregulated inflammatory profile when expressing mutant SOD1 (Beers et al., 2006; Boillée et al., 2006). To create an ALS cellular model based in SOD1 mutations, we transduced the N9 microglia cell line in order to overexpress the hSOD1^WT^ or hSOD1^G93A^ gene. Although some studies have pointed that elevated and not physiological levels of SOD1^WT^ may cause the neurodegeneration and disease onset in the SOD1 ALS mouse model (Wang et al., 2009; Afshar et al., 2017), the transduction process of WT and mutant hSOD1 in our cellular model did not cause any loss of cell viability. Furthermore, N9 hSOD1^WT^ cells presented a higher viability when compared to N9 naïve cells, which may derive from the antioxidant properties of SOD1 protein, thus conferring increased resistance to cell death.

A study using the N9 microglia cell line demonstrated that SOD1 overexpression was able to decrease superoxide and NO production, as well as to attenuate the ability of activated microglia to induce toxicity in cultured neurons (Dimayuga et al., 2007). Here, we observed a decrease in the selected pro- and anti-inflammatory genes evaluated upon hSOD1^WT^ expression, reflecting a deactivation process of the N9 microglia. The same was not observed in the case of hSOD1^G93A^ overexpression, which led to heterogeneous activation and polarization of the cells. In fact, microglia isolated from the spinal cord of the mSOD1 model is characterized by an M1/M2 dichotomy (Chiu et al., 2013), but no clear information was provided regarding the cortical brain microglia (Righi et al., 1989), as the N9 model used in our study. In addition, a recent study showed that microglia activation leads to increased expression of pro-inflammatory genes that is accompanied by the expression of some anti-inflammatory genes, as well (Lively and Schlichter, 2018). Interestingly, when looking at the miRNA profile of transduced cells, miR-146a was only found increased in N9 hSOD1^WT^ cells, which may determine the inhibition of the inflammatory response by targeting the NF-κB pathway with consequent downregulation of the alarmin S100B and the pro-inflammatory cytokines. Since NF-κB activation is necessary for the induction of several miRNAs, such as miR-125b and miR-21, this can explain the reduced levels of these miRNAs in N9 hSOD1^WT^ cells. On the other hand, their downregulation in hSOD1^G93A^ N9 microglia may reveal that cells became reactive and lose their ability to counteract an additional stress stimulus, considering the anti-inflammatory role of these miRNAs (Tili et al., 2007; Ma et al., 2011; Cardoso et al., 2016). In accordance with these findings, N9 hSOD1^G93A^ cells revealed an overexpression of the RAGE receptor. Such result may derive from the activated inflammatory cascade subsequent to the binding of HMGB1 to RAGE, based on the elevated gene expression of such alarmin in these cells, with consequent increased expression of TNF-α, and IL-1β, as here observed by us. Indeed, increased expression of pro-inflammatory mediators in SOD1^G93A^ mice has already been described (Hensley et al., 2006; Jeyachandran et al., 2015) and mutant SOD1^G93A^ microglia was reported to be more neurotoxic than WT microglia, also due to the increased production of ROS and TNF-α pro-inflammatory cytokine (Liu et al., 2009). Additionally, WT and mutated SOD1 overexpression cause a decrease in Fizz1 and IL-10 expression genes, suggesting a delayed response of the transduced microglia toward repair mechanisms during neuroinflammation.

Furthermore, in the mutant SOD1 mouse model, as well as in fALS and sALS patients, miR-155 was found to be elevated and its targeting revealed to restore the dysfunctional microglia and to attenuate disease progression in the ALS mouse model (Koval et al., 2013; Butovsky et al., 2015; Cunha et al., 2018). In N9 hSOD1^G93A^ microglia, despite the similar endogenous levels of miR-155 and miR-146a relatively to naïve cells, these miRNAs were found to be shuttled into exosomes, similarly to our previous observations on N9 microglia polarization by LPS (Cunha et al., 2016). Our findings in hSOD1^G93A^ overexpressing cells highlight the exacerbated expression of inflammatory genes indicating that cells carrying such mutation are more reactive than the N9 hSOD1^WT^ microglia, probably influencing the surrounding cells and contributing to an overall inflammatory environment. In accordance, gene expression analysis in exosomes from mutated cells also revealed that HMGB1 and SOD1 are transported as part of their cargo, together with miR-155 and miR-146a, contributing to the dissemination of inflammatory mediators and aggravating the neuroinflammation status. These results are supported by previous studies showing that mutant/misfolded SOD1 is released into exosomes and is propagated to different cells, while also inducing exosome formation and release from cells (Basso et al., 2013; Grad et al., 2014; Silverman et al., 2016). HMGB1 is described as being secreted either in apoptotic bodies (Bell et al., 2006; Buzas et al., 2014), or in cell-derived exosomes (Sheller-Miller et al., 2017), but never mentioned to be carried in microglia-derived exosomes, as we now report. Although MFG-E8 is not a typical pro-inflammatory mediator, activated microglia upregulate MFG-E8, which has been related with the phagocytosis of viable neurons (Fricker et al., 2012) and with the production of pro-inflammatory cytokines (Liu et al., 2013). However, MFG-E8 role appears to be dose-dependent, since it is also proposed that a high concentration of MFG-E8 blocks the binding between apoptotic cells and microglia/macrophages, thus leading to the inhibition of phagocytosis (Yamaguchi et al., 2008). This may indicate that, in an inflammatory state, in which these cells appear to be, N9 hSOD1^G93A^ microglia can either cause neuronal death or be unable to restore the homeostatic balance, contributing to ALS pathogenesis.

Overexpression of hSOD1^WT^ and hSOD1^G93A^, although useful to understand ALS underlying mechanisms, are not physiological models. In this sense, only the transduced cells with GFP stimulated with LPS reproduced the M1 polarization found in the naïve cells after 24 h treatment (Cunha et al., 2016), while a defective stimulation and depressed resolving capacity after LPS pro-inflammatory activation was found in the transduced N9 hSOD1^WT^ and hSOD1^G93A^ microglia. We may then hypothesize that these microglia may become inefficient protectors of the brain against endogenous damage and pathogens. Actually, when compared with the response of the naïve cells towards LPS, hSOD1^WT^ and hSOD1^G93A^ N9 microglia clearly revealed a decrease in the majority of pro- and anti-inflammatory mediators. Moreover, intracellular inflammatory-associated miRNAs were downregulated, including miR-155 and miR-146a, which are both involved in the NF-κB pathway, by being upregulated upon inflammatory stimulus, and by acting as a negative feedback regulator, respectively (Mann et al., 2017). Though SOD1 overexpression was indicated to reduce neurotoxic inflammatory signaling in microglia (Dimayuga et al., 2007), we could still observe an increased exosomal content in miR-155 meaning that immunomodulatory effects were conserved, a finding not observed in exosomes from the mutated N9 microglia. Instead, their enrichment in miR-125b was shown to account for decreased MN survival in ALS, once miR-125b was found to be neurotoxic and to be upregulated in the lumbar spinal cord of SOD1^G93A^ mice (Parisi et al., 2016).

After concluding that hSOD1^G93A^ N9 microglia show upregulated pro-inflammatory markers, downregulated resolving genes, and exosomal enriched cargo in HMGB1 and SOD1, we tested the immunoregulatory properties of GUDCA and VS in rescuing the dysfunctional phenotype of the mutated microglia towards a more neuroprotective one, based on previous results (Vaz et al., 2015; Falcão et al., 2017). Our data demonstrate that both GUDCA and VS promote inflammatory resolution leading to a reduction of TNF-α, IL-1β, HMGB1, S100B and miR-155 expression, corroborating their anti-inflammatory properties (Fernandes et al., 2007; Vaz et al., 2015; Ko et al., 2017) in other cells and disease models. Interestingly, we observed a significant increase of the anti-inflammatory cytokine IL-10, together with miR-21 and membrane receptors RAGE and TLR-4 only in GUDCA-treated cells. This is not without precedent, since GUDCA was demonstrated to inhibit the production of TNF-α and IL-1β in astroglial cells by preventing the maturation of these cytokines and their consequent release in an experimental model of jaundice, thus preventing astroglial reactivity (Fernandes et al., 2007). Also, miR-21 was reported to promote an anti-inflammatory response by increasing IL-10 production (Sheedy et al., 2010). In this way, GUDCA may be acting through the activation of the receptor TLR-4, leading to the expression of this miRNA that, consequently, promotes an anti-inflammatory response. The decrease of miR-155 encapsulation in exosomes by GUDCA may further concur to attenuate the stimulation produced by the hSOD1^G93A^ overexpression in N9 cells. Regarding VS, similarly to what we observed in N9-microglia exposed to Aβ peptide (Falcão et al., 2017), this compound showed its anti-inflammatory properties by reducing miR-155, HMGB1 and IL-1β, as well as MMP-2 and MMP-9 activation. Additionally, VS increased the cellular expression of miR-146a and decreased that of miR-155, probably as a consequence of NF-κB pathway inhibition and its immunoregulatory properties (Su et al., 2016). Furthermore, VS significantly promoted the expression of MFG-E8 that drives microglia phagocytosis (Liu et al., 2013) and the shuttle of miR-21 into exosomes in hSOD1^G93A^ N9 microglia, which was reported to negatively regulate the secondary inflammatory response of microglia (Wang et al., 2018) by acting in recipient cells. The inhibition of the pro-inflammatory response by miR-146a upregulation may also favor the specific downregulation of MMP-2 and MMP-9 (Könnecke and Bechmann, 2013), potentially resulting in the restoration of immune homeostasis and highlighting VS as a microglia-targeted candidate in ALS. We may then conclude that, although through different mechanisms of action, both GUDCA and VS show beneficial therapeutic properties for immunomodulation towards neuroprotection in ALS. Importantly, because each of the compounds showed specificity to unique microglia targets, that may open new possibilities to modulate precise microglia subtypes.

**Figure 8:**
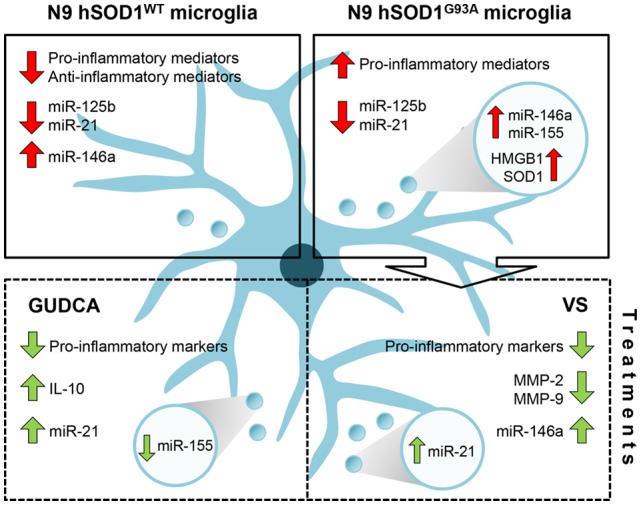
Schematic representation of the dysregulated inflammatory-associated mediators and immunomodulation benefits by GUDCA or VS in N9 cells transduced with hSOD1^WT^ and hSOD1^G93A^ protein. Overexpression of hSOD1^WT^ causes a downregulation of pro- and anti-inflammatory genes in N9 cells and upregulation of miR-146a. In contrast, hSOD1^G93A^ transduction causes increased gene expression of pro-inflammatory mediators, thus leading to an activated microglia population. Both transduced cells reveal a decrease of miR-125b and miR-21. To note that the release of exosomes containing miR-155 and miR-146a, as well as HMGB1 and SOD1 by N9 hSOD1^G93A^ cells may determine the propagation of inflammation. Treatment with GUDCA or VS effectively modulates the gene expression of pro-inflammatory molecules, together with the increase of anti-inflammatory miRNAs. GUDCA increases IL-10 and cellular miR-21, with a substantial reduction of exosomal miR-155, while VS decreases MMP-2 and MMP-9 activation and determines the shuttle of miR-21 into exosomes. In sum, GUDCA and VS are relevant immunoregulators of the dysfunctional microglia in mutated superoxide dismutase 1 (mSOD1) ALS, and their differential target specificity may be envisaged as very promising to recover specific disease-related subtypes. GUDCA, glycoursodeoxycholic acid; HMGB1, high mobility group box protein 1; miR, microRNA; MMP, matrix metalloproteinase; SOD1, superoxide dismutase 1; VS, dipeptidyl vinyl sulfone.

Overall, and as schematically represented in Figure 8, our findings clarify the importance of hSOD1^WT^ in the host cell, by having calming and antioxidant effects. Besides inducing the expression of pro-inflammatory genes, hSOD1^G93A^ protein decreased the expression of anti-inflammatory genes, thus increasing the reactivity of the cell. In addition, by decreasing microglia repairing ability in damaging conditions, the expression of the mutated protein may further contribute to compromised homeostatic balance and failure in neuroregenerative processes. Finally, we propose that GUDCA and VS are potential pharmacological approaches in single or combined therapeutic strategies for ALS, due to their multi-target effects and active microglia immunomodulation.

## Author Contributions

DB and AV conceived and designed the experiments. AV and CC implemented and optimized the transduction procedures. RM supervised the chemical synthesis of dipeptidyl vinyl sulfone. AV, SP, CE and LC carried out the experiments. SP, AV and DB analyzed the data and wrote the manuscript. DB critically reviewed the content and approved the final version for publication.

## Conflict of Interest Statement

The authors declare that the research was conducted in the absence of any commercial or financial relationships that could be construed as a potential conflict of interest.
