# Supplementary material for: Phenotypic Effects of Wild-Type and Mutant SOD1 Expression in N9 Murine Microglia at Steady State, Inflammatory and Immunomodulatory Conditions
Source: Front Cell Neurosci. 2019 Apr 9;13:109. doi: 10.3389/fncel.2019.00109 (PMC6465643; doi:10.3389/fncel.2019.00109)
Supplement: Supplementary file 1 [file Data_Sheet_1.docx]

Supplementary Material

Article Title

**Ana Rita Vaz, Sara Pinto, Catarina Ezequiel, Carolina Cunha, Luís A Carvalho, Rui Moreira, Dora Brites^*^**

*** Correspondence:** Corresponding Author: dbrites@ff.ulisboa.pt

**Supplementary Materials and Methods**

**N9 cell line transduction: Lentiviral production and generation of N9 cells expressing GFP protein**

We used the N9 microglial cell line as described in the manuscript. Lentiviral particles were produced by co-transfections of HEK293T cells with the packaging plasmids pGal-pol and pRev, the envelope plasmid pVSV-G and the lentiviral expression vector LentiGFP (Addgene, Cambridge, USA).

**Morphological characterization**

For immunofluorescence detection, N9 microglia cells were fixed for 20 min with freshly prepared 4% (w/v) paraformaldehyde in PBS and an immunocytochemical technique was performed as usually in our lab for these cells ([Cunha *et al.*, 2016](#_ENREF_21)). N9 microglia were immunostained with rabbit anti-ionized calcium-binding adaptor molecule 1 (Iba1) (1:250, #019-19741, Wako), and nuclei were counterstained with Hoechst 33258 dye (blue). UV and fluorescence images of ten random microscopic fields (original magnification: 400X) were acquired per sample using an AxioCam HR camera adapted to an AxioScope A1® microscope (Zeiss, Germany), and Zen 2012 (blue edition, Zeiss) software. The cell area, perimeter and Feret’s diameter were determined using the ImageJ software.

**MTS assay**

Cellular reduction of MTS [3-(4,5-dimethylthiazol-2-yl)-5-(3-carboxymethoxyphenyl)-2-(4- sulfophenyl)-2H-tetrazolium] is an enzymatic reaction that occurs in functional cells, being considered a marker of cell viability. Cells were incubated for 1 h, at 37°C, with a combination of MTS and phenazine methosulfate (PMS) in cell medium. Absorbance was red at 490 nm using a microplate reader (Bio-Rad Laboratories; Hercules, CA, USA). For each experiment, the mean value of absorbance obtained from control conditions was considered as 100% of cell functionality ([Falcão *et al.*, 2017](#_ENREF_17)).

**Microglial phagocytosis assay**

To evaluate the phagocytic ability of transduced and non-transduced N9 microglia, cells were incubated with pHRodo *E.Coli* particles for 2 h at 37°C and fixed with 4% (w/v) paraformaldehyde in PBS. Nuclei were counterstained with Hoechst 33258 dye (blue). Fluorescence was visualized using an AxioScope.A1 coupled with an axioCam HR camera (Carl Zeiss). UV and fluorescence images of ten random microscopic fields (original magnification: 400X) were acquired per sample using an AxioCam HR camera adapted to an AxioScope A1^®^ microscope (Zeiss, Germany), and Zen 2012 (blue edition, Zeiss) software. The number of beads ingested per cell was counted and distributed by intervals to determine the condition with the highest phagocytic capacity. At least 100 cells were counted for each independent condition ([Cunha *et al.*, 2016](#_ENREF_14)).

**Supplementary Figures Captions**

**Supplementary Figure 1 – Overexpression of human SOD1 in N9 microglia promotes morphological alterations although with no negative impact on their phagocytic ability and cell viability.** Morphological analysis was performed by immunocytochemistry using anti-Iba1 (in red). Nuclear staining was achieved with Hoechst dye (in blue). Representative results of one experiment are shown (A). Quantitative assessment of cell area (B), perimeter (C) and Feret’s diameter (D) was performed using the program ImageJ. Scale bar represents 20 μm. Evaluation of cell relative viability was performed with a mixture of MTS/PMS and percentage of viable cells was calculated considering naïve cells as 100% (E). Phagocytosis was evaluated by the capacity of cells to engulf beads after transduction. Cells were incubated with pHRODO beads and the number of beads in each cell was counted (F). Results are mean (± SEM) from at least three independent experiments. **p<0.01, *p<0.05 *vs*. N9 naïve cells; ^$^p<0.05 *vs*. N9 hSOD1^WT^ cells; one-way ANOVA (Bonferroni *post-hoc* correction).

**Supplementary Figure 2 – Transduction of N9 cells with GFP does not influence the levels of pro-/anti-inflammatory markers as well as miRNA associated with inflammation, neither cellular ability to respond towards the pro-inflammatory stimulus with lipopolysaccharide (LPS).** Naïve N9 and cells transduced with GFP were treated or not with LPS (300 ng/ml) as indicated in methods. Cellular mRNA expression of pro-inflammatory markers (A), anti-inflammatory markers (B) and inflammatory microRNAs (miRNA, miR, C) was evaluated by quantitative Real-Time PCR (qRT-PCR). The dashed line represents the average value (N9 naïve cells). Results are mean (± SEM) from three independent experiments. *p<0.05 and **p<0.01 *vs*. N9 naïve microglia; ^&^p<0.05 and ^&&^p<0.01 *vs*. N9-GFP microglia; one-way ANOVA (Bonferroni *post-hoc* correction).

**Supplementary Figure 3 –** **GUDCA and VS compounds do not cause changes in cell viability.** Evaluation of cell relative viability was performed with a mixture of MTS/PMS, after 48 h of incubation with glycoursodeoxycholic acid (GUDCA) and vinyl sulfone (VS). Percentage of viable cells was determined considering N9 hSOD1^G93A^ cells as 100%. Results are mean (± SEM) from at least three independent experiments.

**Supplementary Tables**

**Table S1 –** List of primer sequences used in qRT-PCR (gene and microRNA expression).

| **Gene** | **Forward Primer Sequence** | **Reverse Primer Sequence** |
| --- | --- | --- |
| *Arginase 1* | 5’-CTTGGCTTGCTTCGGAACTC-3’ | 5’-GGA GAA GGC GTT TGC TTA GTT C-3’ |
| *Fizz1* | 5’-GCCAGGTCCTGGAACCTTTC-3’ | 5’-GGAGCAGGGAGATGCAGATGAG-3’ |
| *HMGB1* | 5’-CTCAGAGAGGTGGAAGACCATGT-3’ | 5’-GGGATGTAGGTTTTCATTTCTCTTTC-3 |
| *IL-1β* | 5’-CAGGCTCCGAGATGAACAAC-3’ | 5’-GGTGGAGAGCTTTCAGCTCATA-3’ |
| *IL-10* | 5’-ATGCTGCCTGCTCTTACTGA-3’ | 5’-GCAGCTCTAGGAGCATGTGG-3’ |
| *iNOS* | 5’-ACCCACATCTGGCAGAATGAG-3’ | 5’-AGCCATGACCTTTCGCATTAG-3’ |
| *MFG-E8* |  |  |
| *RAGE* | 5’-CTGGTGGGACTGTGACCTTG-3’ | 5’-TCTGCCTGTCATTCCTAGCTC-3’ |
| *S100B* | 5’-GAGAGAGGGTGACAAGCACAA-3’ | 5’-GGCCATAAACTCCTGGAAGTC-3’ |
| *SOCS1* | 5’-CACCTTCTTGGTGCGCG-3’ | 5’-AAGCCATCTTCACGCTGAGC-3’ |
| *SOD1* |  |  |
| *TLR-4* | 5’-ACCTGGCTGGTTTACACGTC-3’ | 5’-GTGCCAGAGACATTGCAGAA-3’ |
| *TNF-α* | 5’-TACTGAACTTCGGGGTGATTGGTCC-3’ | 5’-CAGCCTTGTCCCTTGAAGAGAACC-3’ |
| *β-actin* | 5’-GCTCCGGCATGTGCAA-3’ | 5’-AGGATCTTCATGAGGTAGT-3’ |

| **microRNA** | **Target Sequence** |
| --- | --- |
| hsa-miR-21-5p | 5’- UAGCUUAUCAGACUGAUGUUGA-3’ |
| hsa-miR-125b-5p | 5’-UCCCUGAGACCCUAACUUGUGA-3’ |
| hsa-miR-146a-5p | 5’-UGAGAACUGAAUUCCAUGGGUU-3’ |
| mmu-miR-155-5p | 5’-CTCAGAGAGGTGGAAGACCATGT-3’ |
| SNORD110 | Reference gene |
| U6 snRNA | Reference gene |
| RNU1A1 | Reference gene |
