# Supplementary figures and images for: Phenotypic Effects of Wild-Type and Mutant SOD1 Expression in N9 Murine Microglia at Steady State, Inflammatory and Immunomodulatory Conditions
Source: Front Cell Neurosci. 2019 Apr 9;13:109. doi: 10.3389/fncel.2019.00109 (PMC6465643; doi:10.3389/fncel.2019.00109)

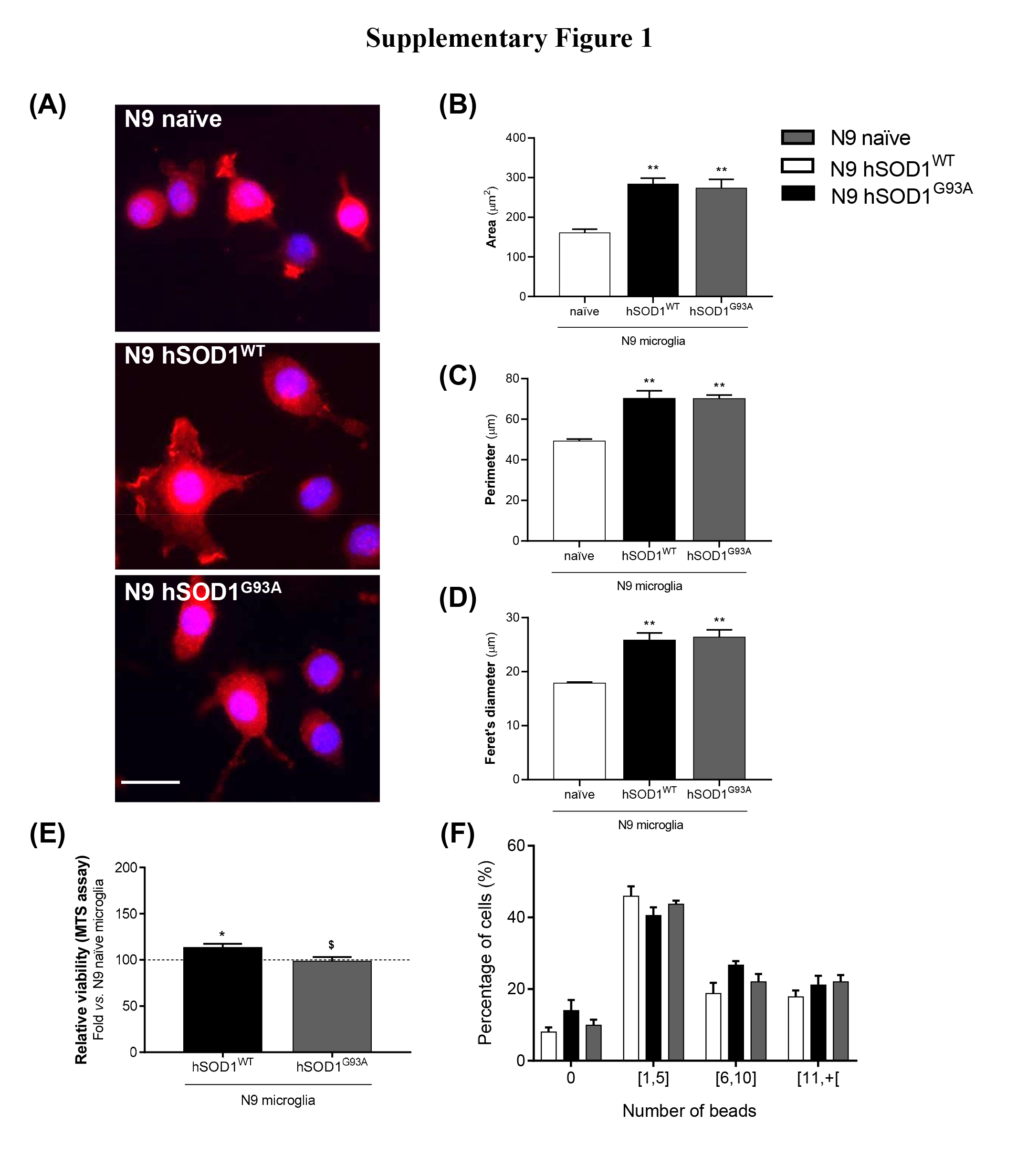

Supplement: Supplementary file 2 [file Image_1.TIF]

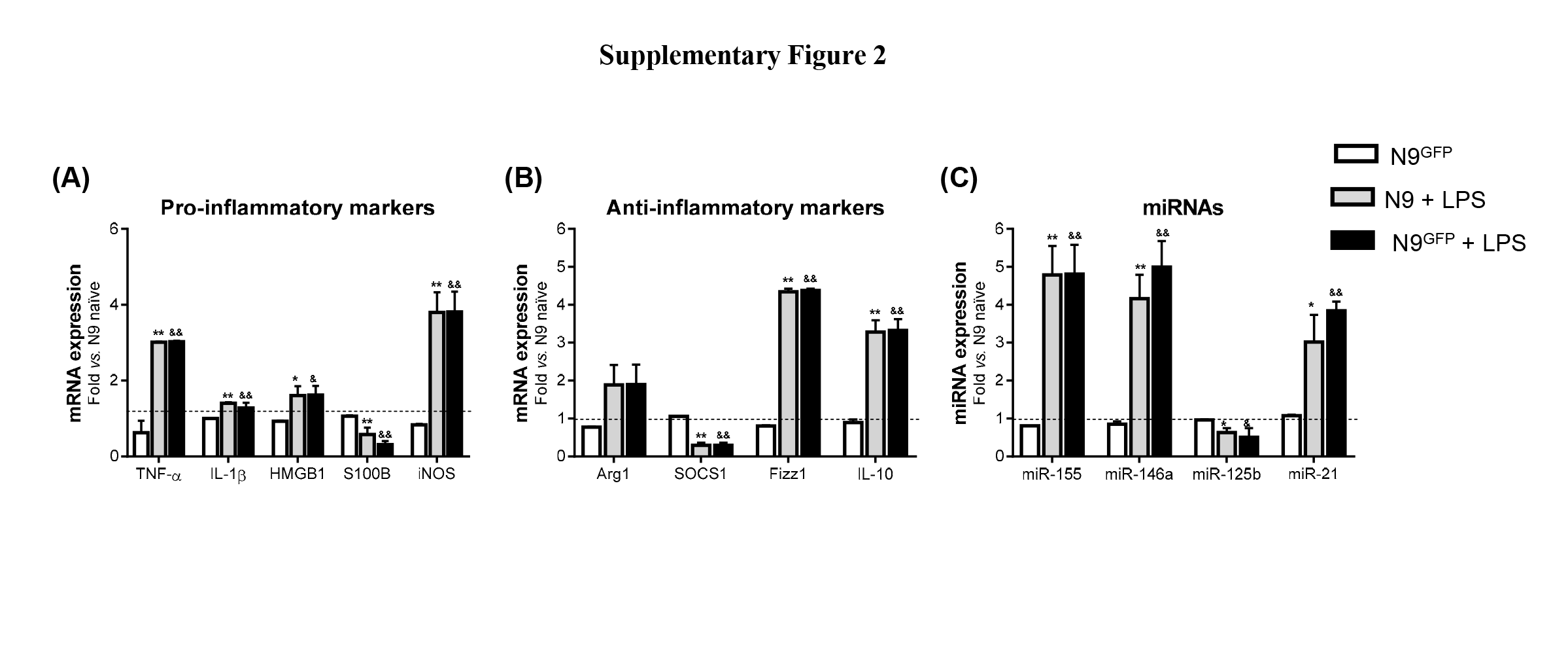

Supplement: Supplementary file 3 [file Image_2.TIF]

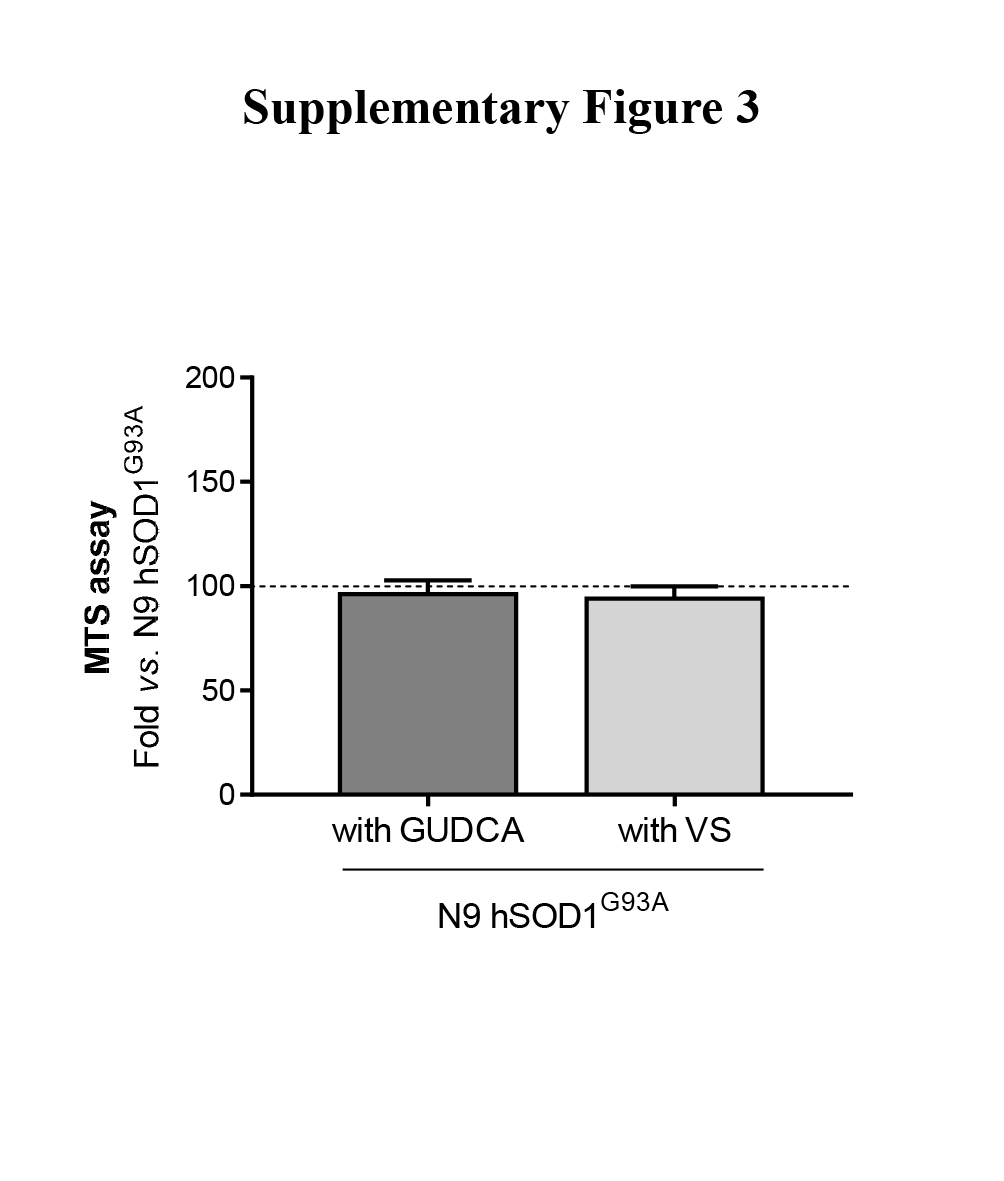

Supplement: Supplementary file 4 [file Image_3.TIF]
